# Correction to Robinson, Whitt, and Jones (2017)

**DOI:** 10.1037/xan0000142

**Published:** 2017-06-05

The article “Familiarity-Based Stimulus Generalization of Conditioned Suppression” by Jasper Robinson, Emma J. Whitt, and Peter M. Jones (*Journal of Experimental Psychology: Animal Learning and Cognition,* 2017, Vol. 43, No. 2, pp. 159–170. http://dx.doi.org/10.1037/xan0000134) was incorrectly published under American Psychological Association copyright. The authors should have retained copyright of this article under the Creative Commons Attribution License. The online version of this article has been corrected.

